# Disability and health-related quality of life in outpatients with generalised anxiety disorder treated in psychiatric clinics: is there still room for improvement?

**DOI:** 10.1186/1744-859X-10-7

**Published:** 2011-03-14

**Authors:** Julio Bobes, Luis Caballero, Inma Vilardaga, Javier Rejas

**Affiliations:** 1 Psychiatry Department - Oviedo University, Centro de Investigación Biomédica en Red de Salud Mental (CIBERSAM), Oviedo, Asturias, Spain; 2 Psychiatry Department, 'Puerta de Hierro' Hospital, Madrid, Spain; 3 Biometrics Department, European Biometric Institute, Barcelona, Spain; 4 Health Outcomes Research, Medical Business Unit, Pfizer España, Alcobendas, Madrid, Spain

## Abstract

**Objective:**

We assessed the impact of generalised anxiety disorder (GAD) on disability and health-related quality of life in outpatients treated in psychiatric clinics via a secondary analysis conducted in 799 patients from a cross-sectional study of prevalence of GAD in psychiatric clinics.

**Methods:**

Patients were allocated into two groups: follow-up (15.7%) and newly diagnosed patients (84.3%), and were administered the Hamilton Anxiety Scale (HAM-A), Clinical Global Impressions Scale (CGI), Sheehan Disability Scale (SDS), and 36-item short form structured quality of life questionnaire (SF-36) scales.

**Results:**

The newly diagnosed group showed higher significant intensity of anxiety (56.9% vs 43.0% (HAM-A >24)), psychiatrist's CGI Severity (CGI-S) scores (4.2 vs 3.7), and perceived stress according to SDS (5.7 vs 5.2). They also showed lower scores in mental health-related quality of life: 25.4 vs 30.8. Statistical differences by gender were not observed. GAD was shown to have a significant impact on patient quality of life and disability, with a substantial portion having persistent, out of control symptoms despite treatment.

**Conclusions:**

These results suggest that there is still room for improvement in the medical management of patients with GAD treated in psychiatric clinics.

## Introduction

The prevalence of generalised anxiety disorder (GAD) has markedly increased over the last few years [1]. Moreover, given its chronicity of symptoms and associated disability, this disorder implies high direct costs for national healthcare systems, as well as indirect costs due to related absenteeism and lost wages [1-6]. The patient suffers anxiety not only within the psychical sphere, but also through somatic manifestations, with GAD being a comorbid condition in many disorders [7-9]. GAD is a generalised and persistent condition with a trend towards chronicity, with alternating improvement and worsening phases that are generally related to environmental stress situations [10,11]. Several epidemiological studies detailing the prevalence in the general population have been performed, with results in Spain ranging between 1.2% and 2.3% [12]. However, in neighbouring countries such as France, life prevalence may be as high as 6.8% [13]. GAD is associated with different comorbidities, which may be both psychiatric and organic [14,15]. In primary care settings, the prevalence is usually higher, ranging between 8% and 20% of the patients treated [5,9,16,17]. Its clinical impact often produces a series of consequences on the lives of patients and those who surround them [4-6]. Patients report deterioration in their physical and intellectual ability, emotional state, personal relationships and career development, so that they require integrated intervention strategies, including a suitable therapeutic approach and/or behavioural therapy [18-21]. In this sense, it is necessary to emphasise that, in the environment of psychiatry in Spain, it is thought that only one-third of patients receive appropriate treatment based on clinical guidelines or expert criteria [22].

The negative effects of GAD on health-related quality of life are among the greatest of the serious mental disorders, particularly when compared with those described in major depression, a disorder known to have a high incapacitating potential and to be health-resource consuming [1-3,23-25]. Aspects involving subjective perceptions of health improve when patients have undergone the right treatment, as is shown in controlled clinical studies [26]. In contrast, so far there are few specific published studies assessing the control of symptoms in routine clinical practice in GAD; therefore, a better knowledge of this condition, its management in clinical practice and patient responsiveness to medical interventions, evaluated from both a clinical and self-perceived approach, is required [27,28].

Treatment of GAD has traditionally focused on anxiolytic drugs, either in monotherapy or in combination. Pharmacological treatment of GAD has usually consisted of benzodiazepines (BZDs), buspirone, and imipramine. At present, selective serotonin reuptake inhibitors (SSRI) are the treatment of choice for GAD. Venlafaxine, a serotonin and noradrenaline reuptake inhibitor (SNRI), is also used for treatment of GAD [29], and recently it has been shown that the SSRI paroxetine is an effective treatment for GAD as well [30]. However, despite the efficacy shown by these drugs in the treatment of GAD, and regardless of the recommendations established in many countries for their use, it is necessary to incorporate the clinical control of the patient's symptoms into daily medical practice. As a complement to the clinical criterion, it is also very useful to know the patient's own perception of his/her health and how he/she adapts to the environment. The objective of this secondary analysis using data from a nationwide epidemiological study, whose purpose was to determine the clinical ambulatory prevalence of GAD in psychiatric clinics [31], was to assess how GAD impacts patient disability and quality of life.

## Materials and methods

### Design and procedure for patient selection

The study population was comprised of patients of both sexes selected nationwide in Spain, recruited by a total of 312 psychiatrists, as part of the LIGANDO study [31], an epidemiological study whose purpose was to determine the clinical ambulatory prevalence of the GAD in psychiatric clinics. The participating investigators were selected in random clusters, weighted by population rates in each geographic region from a database of physicians participating in clinical research. Psychiatrists had to have participated in previous psychiatric research projects in the field of anxiety. A cross-sectional multicentre and observational design was chosen, performed using the data from patients seen in outpatient psychiatric visits (hospitals and mental health centres) during the period from October to December 2006. In order to obtain the relevant information on each patient and not to interfere with routing clinical practice, the study was designed to obtain all information at one face-to-face visit only. Using a non-probabilistic consecutive sampling method, each investigator registered the data (reason for the visit, diagnosis, age, and gender) of the first 75 patients seen for any reason, for a maximum period of 3 months. The first three patients with a confirmed clinical diagnosis of GAD who met the selection criteria were included in the study. The information sheet with the detailed study objectives and procedures was handed out to these patients only and they were asked to give their informed consent to participate in the study. In the end, the patients enrolled in the study were (a) of either sex and aged over 18 years, (b) had a diagnosis of GAD according to International Statistical Classification of Diseases and Related Health Problems 10th Revision (ICD-10) criteria [32], (c) had been treated at a mental health centre or outpatient psychiatric clinic, and (d) the patient himself/herself had given his consent to participate. Patients with severe psychiatric disorders (such as acute schizophrenia) or severe cognitive impairment diagnosed in the usual manner [33], or who presented difficulties or inability to answer the health questionnaires written in Spanish were excluded.

The sample size was determined by the protocol of the epidemiological study within which the present study was conducted. Based on normally distributed proportions, assuming an expected prevalence in specialised care of 9.1%, on a bilateral test (2-tailed test), with a 95% confidence interval, for an infinite sample and an expected accuracy of 2%, the sample size calculation should not be lower than 790 patients with GAD. On a population-wide basis, assuming a percentage of loss of 15% and prevalence in the community of 5%, a minimum requirement of 303 psychiatrists and 18,000 patients was estimated.

### Operational variables and main outcomes

During the screening visit, patients were separated into two groups: new cases (newly diagnosed patients) and patients already diagnosed with GAD who were returning for control tests of the progression of their symptoms (follow-up patients). The main outcomes of the study were obtained by means of a data collection questionnaire. These consisted of general and concomitant treatment, clinical impact, disability, and self-perceived health. The general and medication-related variables were age (continuous and ranges of values), gender, body mass index (BMI, kg/m^2^), time of diagnosis and delayed time to diagnosis (from the onset of symptoms to confirmation of GAD by diagnosis, in years). Current and prior medications received as treatment for GAD were categorised by The Anatomical Therapeutic Chemical Classification System [34] in five groups: (a) benzodiazepines ((sedative and hypnotic), alprazolam, bentazepam, bromazepam, clorazepate dipotassium, clonazepam, diazepam, halazepam, lorazepam, lormetazepam, and ketazolam), (b) selective serotonin/noradrenergic reuptake inhibitors (citalopram, duloxetine, escitalopram, fluoxetine, fluvoxamine, mirtazapine, paroxetine, sertraline, venlafaxine, and trazodone (SSRI/SNRI)), (c) tricyclic antidepressants (amitriptyline, clomipramine, imipramine, and maprotiline), (d) antiepileptics (gabapentin, pregabalin, and topiramate), and (e) other groups and drug combinations (amitriptyline/medazepam, diazepam/sulpiride, flupentixol/melitracen, olanzapine, sulpiride, and zolpidem).

For the clinical impact assessment, two scales were administered: the Hamilton Anxiety Scale (HAM-A) [35] and Clinical Global Impressions Scale (CGI) [36]. The degree of patient disability was measured by means of the Sheehan Disability Scale (SDS) [37], while self-perceived health was assessed with the 36-item short form structured quality of life questionnaire (SF-36) [38]. All of the scales were used in their validated Spanish versions. The classification of intensity of anxiety according to total score was: no anxiety: score from 0 to 9; mild: from 10 to 15; moderate: from 16 to 24; and intense: over 24. The HAM-A items related to insomnia (HAM-A_insomnia_), cognition (HAM-A_cognition_) and depressed mood (HAM-A_mood_) were analysed independently. The aim of the CGI scale is to assess patient responsiveness to treatment. It is composed of two subscales: CGI Severity (CGI-S) and CGI Improvement (CGI-I). In this study, the CGI-S evaluated was assessed by the psychiatrist during the visit/interview. The CGI-S consists of only one item that assesses severity using a Likert scale of eight values, ranking from 1 (normal, not ill) to 7 (extremely ill), with 0 indicating not evaluated.

### Confidentiality of information, security, and statistical analysis

Information was treated with the utmost levels of confidentiality, in accordance with applicable national data protection legislation. The ethical principles of the Declaration of Helsinki were followed by all the investigators. Although this study did not have describing adverse reactions as an objective, the investigator had the obligation to report any serious adverse reaction he encountered during the study. The study protocol was approved by the Research Ethics Committee of Hospital Universitario de Asturias, Oviedo.

The main outcomes were patient's responses on scales of health-related quality of life (HRQoL) (SF-36) and disability (SDS), and also on the CGI. Scores on the CGI and SDS scales were evaluated as absolute values observed, while scores on the SF-36, by each of its domains and summary subscales, are expressed in standardised metrics (0 to 100) and in typified scores for the Spanish reference population. Typified scores are expressed in Z scores, calculated with the following formula: Z score = (observed value - arithmetic mean of the reference population)/standard deviation in reference population, this allowing us to estimate the extent to what patients in this study separate from the normal population in Spain. Also, scoring on the HAM-A scale was evaluated calculating total scoring and punctuation on the HAM-A subscales, as absolute values and classified in levels of anxiety: intense (> 24), moderate (16-24), mild (10-15) and without anxiety (0-9). We also calculated the global numbers and proportions of patients in the subgroups who presented a level from marked to severe (≥3) on individual items of the HAM-A related to cognition, insomnia, and depression, and from moderate to intense for the pain item of SF-36.

A descriptive statistical one-way analysis with values for mean, standard deviation and 95% confidence intervals (CI) was performed; normal distribution was checked with the Kolmogorov-Smirnov test. For the main analysis of this study, patients were classified into two groups: follow-up patients (those previously diagnosed and receiving treatment at the time of the study) and newly diagnosed patients (patients who were diagnosed with GAD at the baseline study visit). In the bivariate analysis, a χ^2 ^test, Student's *t *test for independent groups, and analysis of covariance (ANCOVA) were also used to adjust for demographic confounders. A Bonferroni correction procedure was applied for multiple pairwise comparisons. SAS software version 8 was used (SAS, Cary, NC, USA), and the statistical significance was set at *P *< 0.05.

## Results

From an initial selection of 20,347 patients treated by 312 psychiatrists in mental healthcare centres throughout Spain during the epidemiological study period in 2006, information was obtained for 19,962 patients. Of those, 13.7% (n = 2.743; CI 13.2% to 14.2%) met the clinical criteria for GAD according to ICD-10 (prevalence). For the present study, a total of 799 patients were selected consecutively. Nine patients could not be assessed (0.9%) for the following reasons: because they were under 18 years old (n = 1), because they had a severe psychiatric condition (n = 6), and due to severe/moderate cognitive deterioration (n = 2). None of the patients refused to complete the questionnaires. Of the patients assessed in this study, 124 were included in the group of patients newly diagnosed with GAD (15.7%) and 666 in the follow-up group (84.3%).

Table 1 describes the general sociodemographic characteristics of patients. The mean age was 44.4 years, and 68.9% were women. Subjects from the follow-up group were older compared with subjects in newly diagnosed group (44.9 vs 41.7 years; *P *= 0.016), and were taking more medications at the time of study visit (2.5 vs 1.8; *P *< 0.001). A total of 72.9% of the patients from the newly diagnosed group were being exposed to benzodiazepines, compared with 47.3% in the follow-up group, *P *< 0.001. Compared with the follow-up group, in the newly diagnosed group there was a greater proportion of patients with intense anxiety symptoms (HAM-A >24); 56.9% vs 43.0%, *P *< 0.001, and a higher score on both the psychiatrist's CGI (4.2 vs 3.7, *P *< 0.001) and the patient's CGI (4.7 vs 4.1, *P *< 0.001). However, the level of disability assessed by the SDS scale shows similar values in both groups, with no statistically significant differences, apart from the perceived stress level, which was higher, although limited in magnitude, in newly diagnosed patients; 5.7 vs 5.2, *P *= 0.032. Newly diagnosed patients had significantly poorer standardised scores (Z values) than follow-up patients on the mental health summary subscale of the SF-36 questionnaire; -2.5 vs -1.8, *P *< 0.001 (Table 2). The standardised means from the SF-36 health-related quality of life questionnaire showed poorer self-perception in physical scales, physical role, emotional role, social role, mental health, and vital scale in the newly diagnosed group, *P *< 0.05 in all cases, with standardised scores (Z values) significantly further from the mean value (Table 3), below or near -2 standard deviations in certain cases, as in the emotional role, social function, and mental health values.

**Table 1:** General characteristics of the studied series, according to newly diagnosis or follow-up patient groups

Characteristics	Total (n = 790, 100.0%)	Newly diagnosed (n = 124, 15.7%)	Follow-up (n = 666, 84.3%)	*P *value
Age (SD), years	44.4 (13.1)	41.7 (13.0)	44.9 (13.1)	0.016

Ranges:				

19-35 years	25.7%	36.8%	23.6%	0.025

36-50 years	40.8%	36.8%	41.7%	

51-65 years	26.0%	19.3%	27.2%	

> 65 years	7.4%	7.0%	7.5%	

Gender (female)	68.9%	63.7%	69.8%	NS

Diagnosis delay (SD), years	2.3 (4.9)	2.2 (4.6)	2.3 (5.0)	NS

Body mass index, kg/m^2^	24.8 (4.0)	24.2 (3.3)	25.0 (4.1)	0.018

Drug intake at time of the study visit (n):				

Average	2.4 (1.2)	1.8 (1.2)	2.5 (1.2)	< 0.001

1	26.0%	55.4%	22.7%	< 0.001

2	33.3%	24.6%	34.3%	

3	21.3%	6.2%	23.0%	

4	14.5%	9.2%	15.1%	

5 or more	4.9%	4.6%	4.9%	

Drug intake at the time of the study visit (class):				

Benzodiazepines	49.3%	72.9%	47.3%	< 0.001

SSRI/SNRI	34.9%	15.3%	36.6%	

Tricyclic antidepressants	9.2%	6.8%	9.5%	

Antiepileptics	2.8%	1.7%	2.9%	

Other medication	3.7%	3.4%	3.7%	

Values are expressed as arithmetic mean (SD).

NS = not significant; SSRI/SNRI = selective serotonin reuptake inhibitors/mixed action selective serotonin reuptake inhibitors or noradrenergics.

**Table 2:** Assessing of clinical impact in two patients groups (newly diagnosed/follow-up), according to different scales used

Scale	Characteristics	Total (n = 790, 100.0%)	Newly diagnosed (n = 124, 15.7%)	Follow-up (n = 666, 84.3%)	*P *value
HAM-A	Intensity of the categorised anxiety				< 0.001

	Intense (> 24)	45.2%	56.9%	43.0%	

	Moderate (16-24)	33.8%	38.2%	32.9%	

	Mild (10-15)	11.3%	4.1%	12.6%	

	Without anxiety (0-9)	9.8%	0.8%	11.4%	

	Subscale for psychic anxiety	12.3 (4.9)	14.2 (3.6)	12.0 (5.0)	< 0.001

	Subscale for somatic anxiety	10.8 (5.2)	12.6 (4.5)	10.4 (5.2)	< 0.001

	Total scoring	23.1 (9.4)	26.8 (7.3)	22.4 (9.6)	< 0.001

CGI	Psychiatrist	3.8 (1.1)	4.2 (0.8)	3.7 (1.1)	< 0.001

	Patient	4.2 (1.3)	4.7 (1.0)	4.1 (1.3)	< 0.001

SDI	Incapacity for work	5.3 (2.7)	5.7 (2.4)	5.3 (2.8)	NS

	Incapacity for social life	5.6 (2.6)	5.9 (2.2)	5.5 (2.6)	NS

	Incapacity for family relationships	5.1 (2.5)	5.6 (2.2)	5.1 (2.6)	NS

	General incapacity (work-social-family)	16.0 (7.0)	17.1 (6.0)	15.9 (7.2)	NS

	Perceived stress	5.3 (2.3)	5.7 (2.1)	5.2 (2.4)	0.032

	Perceived social support	56.1 (26.1)	59.0 (23.9)	55.7 (26.4)	NS

SF-36	Physical health summary index	43.3 (9.0)	43.3 (8.9)	43.3 (9.1)	NS

	Z Punctuation physical health summary index	-0.7 (0.9)	-0.7 (0.9)	-0.7 (0.9)	NS

	Mental health summary index	30.0 (11.4)	25.4 (8.5)	30.8 (11.7)	< 0.001

	Z Punctuation mental health summary index	-2.0 (1.1)	-2.5 (0.8)	-1.9 (1.2)	< 0.001

Values are expressed as mean (SD) or percentages.

CGI = clinical global impression of disease; HAM-A = Hamilton Anxiety scale (HAM-A); NS = not significant SDS = Sheeham Disability Scale (SDS); SF-36 = 36-item short-form health-related quality of life questionnaire.

**Table 3:** Distribution of different dimensions in the 36-item short-form quality of life related questionnaire (SF-36), according to newly diagnosed or follow-up patient groups

SF-36 Dimensions	Total (n = 790, 100.0%)	Newly diagnosed (n = 124, 15.7%)	Follow-up (n = 666, 84.3%)	*P *value
Physical functioning	-0.5 (1.0)	-0.5 (0.9)	-0.5 (1.0)	NS

Physical role	-1.4 (1.1)	-1.6 (1.1)	-1.4 (1.1)	0.011

Emotional role	-1.9 (1.3)	-2.3 (1.0)	-1.8 (1.4)	0.001

Social functioning	-2.1 (1.3)	-2.4 (1.1)	-2.0 (1.3)	< 0.001

Pain	-0.9 (1.0)	-0.9 (1.0)	-0.8 (1.0)	NS

Mental health	-1.5 (0.9)	-1.8 (0.7)	-1.4 (0.9)	< 0.001

Vitality	-1.3 (0.9)	-1.5 (0.8)	-1.3 (0.9)	0.002

Overall health status	-1.4 (0.9)	-1.5 (0.8)	-1.4 (0.9)	NS

Values are expressed as mean Z scores (SD).

NS = not significant.

**Figure 1:**
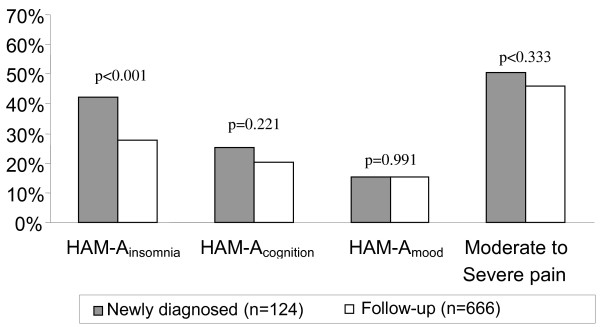
**Relative distribution of sleep disturbances, cognition, depressed mood, and pain on the Hamilton Anxiety scale (HAM-A) and short-form 36 item (SF-36) structured health-related quality of life questionnaire, by study groups (newly diagnosed/follow-up)**.

Newly diagnosed patients presented insomnia (≥3 in HAM-A_insomnia _item) in a greater proportion than follow-up patients; 42.3% vs 27.8%, *P *= 0.0013 (Figure 1), while there were no statistically significant differences found between the two groups in the presence of marked or severe deterioration of cognition (≥3 in HAM-A_cognition _item), depressed mood ((≥3 in HAM-A_depression _item) or presence of pain from moderate to very high as a comorbidity (SF-36_pain_, Figure 1). However, it is noteworthy that 15.5% of the total patients presented depressed mood according to the HAM-A, considerable deterioration of cognition in 21.1% of the patients, or presence of moderate to severe pain in 46.7% of the recruited patients.

It is important to highlight that, by gender, no significant differences were observed in the different questionnaires evaluated (HAM-A, CGI, SDS, and SF-36). In the analysis by age groups (shown in Table 4), and compared with the reference population, patients older than 50 years of age perceive poorer quality of life in physical functioning, physical role, pain, vitality, general health perception scales, and in physical health summary subscale, *P *< 0.05 in all cases.

**Table 4:** Standardised mean scores and Z scores of the patients with generalised anxiety disorder according to age ranges and dimensions from the 36-item short-form quality of life related questionnaire (SF-36)

SF-36 Dimension		Age group, years				
		
		19-35 (n = 200, 25.3%)	36-50 (n = 326, 41.2%)	51-65 (n = 207, 26.2%)	> 65 (n = 57, 7.2%)	*P *value
Physical functioning	Std	78.1 (22.2)	73.7 (22.4)	64.2 (24.3)	58.3 (23.7)	< 0.001

	Z	0.3 (0.9)	0.5 (0.9)	0.9 (1.0)	1.1 (1.0)	< 0.001

Physical role	Std	38.7 (41.4)	36.0 (40.4)	24.7 (33.8)	29.1 (36.6)	0.003

	Z	1.3 (1.2)	1.3 (1.1)	1.7 (1.0)	1.5 (1.0)	0.003

Emotional role	Std	29.6 (37.2)	33.1 (40.9)	26.3 (37.3)	38.1 (42.5)	NS

	Z	2.0 (1.2)	1.8 (1.4)	2.1 (1.2)	1.7 (1.4)	NS

Social functioning	Std	50.6 (26.8)	49.0 (26.2)	45.0 (22.4)	46.4 (24.5)	NS

	Z	2.0 (1.3)	2.1 (1.3)	2.3 (1.1)	2.2 (1.2)	NS

Pain	Std	61.0 (26.3)	55.9 (27.4)	47.0 (24.8)	50.7 (26.1)	< 0.001

	Z	0.6 (0.9)	0.8 (1.0)	1.1 (0.9)	1.0 (0.9)	< 0.001

Mental health	Std	44.5 (19.8)	44.5 (19.5)	40.9 (15.5)	41.6 (16.3)	NS

	Z	1.4 (1.0)	1.4 (1.0)	1.6 (0.8)	1.6 (0.8)	NS

Vitality	Std	41.5 (20.8)	37.7 (20.7)	33.6 (17.5)	34.7 (16.4)	0.015

	Z	1.1 (0.9)	1.3 (0.9)	1.5 (0.8)	1.5 (0.7)	0.015

Overall health status	Std	40.5 (22.0)	36.8 (20.0)	31.7 (16.6)	31.6 (17.3)	0.002

	Z	-1.2 (1.0)	-1.4 (0.9)	-1.6 (0.7)	-1.6 (0.8)	0.002

Physical health summary index	Std	45.9 (8.9)	43.7 (8.7)	40.1 (8.5)	39.0 (9.4)	< 0.001

	Z	0.4 (0.9)	0.6 (0.9)	1.0 (0.9)	1.1 (0.9)	< 0.001

Mental health summary index	Std	29.7 (11.7)	30.3 (12.0)	28.9 (9.9)	31.1 (11.6)	NS

	Z	2.0 (1.2)	2.0 (1.2)	2.1 (1.0)	1.9 (1.2)	NS

Values are expressed as mean (SD).

NS = not significant; Std = standardised scores.

## Discussion

In this study, conducted as part of a clinical prevalence study on GAD, the impact of clinical intensity and the outcome on self-perceived health was specifically investigated for GAD patients treated in psychiatric outpatient clinics in routine medical practice conditions. In addition, the level of control of patient symptoms and the possible coexistence of certain comorbid disorders (intellectual sphere, state of mood, sleep disorders, and pain) were assessed as well. Despite the fact that this type of design represents an appropriate frame for studies to be performed within the specialised healthcare environment and routine clinical practice; it is worth highlighting that this study was conducted in only one visit, so it provides only a 'snapshot' of the patients treated for GAD in an outpatient visit. The lack of randomised patient selection or prospective follow-up made us careful in interpreting the results within the health policy, healthcare provider, and clinical management environments, leading us to take a cautious approach to the external validity of the results [39]. However, an interesting aspect worth stating is that studies with these characteristics provide physicians with information about the patients' situation in 'real world' conditions, and are therefore complementary to the information from the controlled clinical trials, helping with health decision making.

In our study, it was observed that follow-up patients treated at an outpatient visit were older, had a higher BMI, and more previous drug exposure (intake), especially to antidepressants, compared with newly diagnosed patients. A higher body mass index in the follow-up group could be due to a combination of hormonal factors (women), abandon of healthy habits because of the illness, and/or more use of antidepressant medication, when certain drugs are associated with weight gain in patients throughout the course of treatment (paroxetine, for example) [5,9,16,17]. However, there were no statistically significant differences in diagnosis delay in the two groups. The prior exposure to antidepressants and benzodiazepines observed in the newly diagnosed group suggests the possibility of a considerable hidden rate of GAD diagnosis or diagnosis errors. Therefore, these patients, as the mean diagnosis delay value shows, could have been treated for some of their symptoms at a medical care level other than a psychiatrist without reaching a diagnosis of GAD. Our results state that 72.9% of the patients in the newly diagnosed group had received benzodiazepines at some time before this study visit, in comparison with the 47.3% of the follow-up group.

To start with an anxiolytic treatment and to combine it with an antidepressant afterwards could be a clinical criterion, also consistent with the numerous clinical practice guidelines. Nevertheless, it is striking (although it is not specifically detailed in the study) that there was high use of lorazepam, diazepam and bromazepam, which are drugs not considered appropriate according to clinical evidence and international scientific societies [40-42]. This aspect also appears with fluoxetine and citalopram, in both groups analysed. In Spain, according to the indication approved for GAD, it is recommended to use alprazolam, paroxetine, venlafaxine or escitalopram, and those are similar to the recommendations observed in recent reviews published on this topic [15]. However, our findings are consistent with the reviewed studies, where it appears that a GAD diagnosis, according to clinical practice guidelines, is not always in keeping with the most recommended pharmacological treatment [40-43].

It is important to consider that, as expected, the newly diagnosed group shows a higher anxiety intensity (56.9% vs 43.0%), clinical impression seriousness (CGI; 4.2 vs 3.7) and perceived stress (5.7 vs 5.2) compared with the follow-up group, although the magnitude of differences were of moderate clinical significance. The quality of life scores perceived by the patient, as in some placebo controlled trials [44], have also been inferior for metal health domain (25.4 vs 30.8); moreover, typified mean scores (Z scores) on the SF-36 health-related quality of life questionnaire presented poorer self-perception in the following dimensions: physical role, emotional role, social role, mental health, and vitality, in the newly diagnosed group. Even though it was expected that new patients had a poorer state of health and clinical status than follow-up patients, it is surprising that 43% of diagnosed patients, even though receiving the correct treatment, still continue to have an intense level of anxiety (> 24 points on the HAM-A) or that the disability level for everyday routines, including those at work, is similar to that of the newly diagnosed patients. It could be argued that follow-up patients seen at an outpatient visit are those who are not controlled, and therefore must return to their physician to adjust their treatment dose. However, even if this is true, the study recruited consecutive patients, who might include not only those returning for a visit due to a lack of symptom control, but also those following a check-up programme of their GAD.

It should be added that there is a high frequency, according to the corresponding HAM-A scale items, of possible cognitive impairment, sleep disorders, or moderate to intense pain suffered by these patients, which are well known and important in GAD subjects [45-48]. Hence, in the ESEMeD-España study [49], the prevalence-year of back pain or back of the neck pain in general population was 14.7%, while the prevalence in the general population of chronic pain in the Pain in Europe survey was 19% [50], which is in contrast with the 46.7% of patients with GAD suffering pain for the last month in our study. The proportion of patients in this study with possible sleeping disorders, 30.1%, is clearly superior to the proportion observed among the general population [47] and is in keeping with the figures observed by other authors for this disorder [45]. It has been reported in many studies that cognitive functioning in GAD patients may be reduced or deteriorated in some of its components [51-53], and that the cognitive item of the HAM-A improves with specific treatment for GAD [54,55]. However, in our study, we observed a high frequency of patients with considerable impairment on the HAM-A cognitive item (1 out of 5) in spite of being treated with SSRI/SNRI or benzodiazepines, which suggest potential negative effects of certain anxiolytics on cognitive function [53].

The results of this study show the profile of the average patient who visits a psychiatric outpatient clinic under routine medical practice conditions, and they provide important information about the clinical management of these patients. From this, it would be important to consider the need for a continuing training of clinicians in this type of condition, reviewing the criteria for referral to specialists. Also, it should encourage training in prescribing the most suitable therapies according to the possible aetiologic reasons for the condition, aligned with available evidence, and without disregarding the organisational aspects of the waiting list, which can also be negatively perceived by the patients, before arriving at the psychiatric visits. The possible limitations, apart from those previously stated, are derived from the limitations inherent in such studies, for example underestimation of the condition or the possible variability inherent in the routine use of the different clinical screening scales by healthcare professionals. Also, we cannot avoid some bias associated with the method followed to select participants (those with previous experience in epidemiological and/or clinical research). Nevertheless, the data obtained may be an important source of information for healthcare professionals and regulators who are responsible for adopting the appropriate measures at this level of care.

In conclusion, patients who were managed for GAD in an outpatient psychiatric setting within the Spanish Mental Healthcare system presented a considerable impact on their health, both from the clinical perspective and the patient as well. The anxiety symptoms, even treated with anxiolytics/antidepressants, are not perceived as properly controlled yet. These results suggest that there is still room for improvement in medical care for the patient with GAD.

## Competing interests

JR is employed by Pfizer España, who funded this study.

## Authors' contributions

JB, LC and JR participated in the design of the study, interpretation of data, and writing of the manuscript. IV participated in the analysis and interpretation of data and in the preparation of the manuscript. All authors were responsible for literature review and extraction of references.
